# Cell Death and Inflammatory Bowel Diseases: Apoptosis, Necrosis, and Autophagy in the Intestinal Epithelium

**DOI:** 10.1155/2014/218493

**Published:** 2014-07-14

**Authors:** Tiago Nunes, Claudio Bernardazzi, Heitor S. de Souza

**Affiliations:** ^1^Nutrition and Immunology Chair, ZIEL-Research Center for Nutrition and Food Sciences, Technical University of Munich, Weihenstephan, 85354 Freising, Germany; ^2^Serviço de Gastroenterologia & Laboratório Multidisciplinar de Pesquisa, Hospital Universitario, Universidade Federal do Rio de Janeiro, Rua Professor Rodolpho Paulo Rocco 255, Ilha do Fundão, 21941-913 Rio de Janeiro, RJ, Brazil; ^3^D'Or Institute for Research and Education (IDOR), Rua Diniz Cordeiro 30, Botafogo, 22281-100 Rio de Janeiro, RJ, Brazil

## Abstract

Cell death mechanisms have been associated with the development of inflammatory bowel diseases in humans and mice. Recent studies suggested that a complex crosstalk between autophagy/apoptosis, microbe sensing, and enhanced endoplasmic reticulum stress in the epithelium could play a critical role in these diseases. In addition, necroptosis, a relatively novel programmed necrosis-like pathway associated with TNF receptor activation, seems to be also present in the pathogenesis of Crohn's disease and in specific animal models for intestinal inflammation. This review attempts to cover new data related to cell death mechanisms and inflammatory bowel diseases.

## 1. Introduction

### 1.1. Cell Death and Damage Control

The inflammatory process aims to neutralize harmful stimuli as an effort of self-protection [1]. There are basically two types of inflammation: acute and chronic. Acute inflammation comprises the initial response to eliminate the insulting cause without any residual structural or functional damage. It is a temporary phenomenon, which includes later regeneration and complete healing of the involved area [1]. In contrast, when the initial insult persists, the resulting chronic inflammation leads to organ damage, preventing a complete return to homeostasis [2]. In the inflamed gut, both in acute and in chronic inflammation, an effective modulation of the immune response with the subsequent downregulation of inflammation is critical to reduce tissue damage and to promote mucosal healing [3]. In this sense, the programmed cell death machinery is key for the homeostasis reestablishment after an acute or chronic insult, limiting the propagation of the inflammatory stimuli to prevent tissue's loss of function [4].

In vitro studies have demonstrated resistance to apoptosis in lamina propria T cells obtained from the intestinal mucosa of patients with Crohn's disease (CD) [6]. Additional evidence has long supported the association of T cell resistance to apoptosis with altered concentration ratios of Bcl-2 family proteins [7, 8]. In fact, efficacy of anti-TNF-alpha antibodies in inflammatory bowel diseases (IBD) has been associated with apoptosis modulation in lamina propria mononuclear cells, in particular T cells [9, 10], through the induction of the intrinsic apoptotic pathway mediated by Bcl-2 family proteins [11]. Recently, the defective apoptosis of lamina propria T cells in CD was also shown to be related to increased levels of survivin, a family member of the inhibitor of apoptosis proteins (IAP), through the interaction with the chaperone HSP90 [12]. Nevertheless, in the last decade, research in IBD pathogenesis has undergone a progressive shift from the effector arm of inflammation, namely, the adaptive immune system, towards the innate immunity and mechanisms involving the complex interactions between the host and the microbiota.

In recent years, several genome-wide association studies (GWAS) have been undertaken in IBD patients and healthy controls providing an extraordinary new insight into the pathogenesis of these conditions [13–15]. The combined genome-wide analysis of CD and ulcerative colitis (UC) generated a more comprehensive analysis of disease specificity [16]. Currently, the total disease variance explained by heritability in IBD ranges from 7.5% in UC to 13.6% in CD with 110 of 163 loci associated with IBD being found in both diseases [16]. Most known susceptibility genes are involved in autophagy, cellular stress regulation, and microbial pathogen sensing, suggesting that cell death mechanisms might play a key role in the pathogenesis of IBD.

### 1.2. Homeostasis of Intestinal Epithelium

The intestinal epithelium constitutes a specialized single cell layer with absorptive and secretory functions in the interface between the body and the external environment [17]. In the epithelium, enterocytes are responsible for the absorption of nutrients, ions, vitamins, and water and are also involved in the induction of immunological tolerance to ingested peptides [18]. Paneth cells, goblet cells, and enteroendocrine cells comprise the secretory lineage of the intestinal epithelium, having an important role in the intestinal defense against potentially harmful bacteria and the coordination of intestinal functions by hormone secretion [19–21]. In close contact with the epithelium lies the lamina propria, a loose connective tissue in which mesenchymal cells and mucosal immune cells are located.

In the large and small intestine, differentiated enterocytes are removed constantly and replaced by new cells originated by undifferentiated adult intestinal stem cells, localized in the third or fourth position counted from the base of the crypt [22]. These new cells migrate from the base of the crypt to the apical zone of the intestine undergoing maturation. In the apical zone, these cells survive for about 4-5 days prior to being shed into the gut lumen [23]. This single epithelial layer displays a strict balance between cellular proliferation and cell death in order to maintain the intestinal barrier [24]. Importantly, if the epithelium cell death is not strictly regulated, it might result in a barrier defect with subsequent microbial invasion and inflammation. In this regard, previous studies have shown that epithelial proliferation and turnover are accelerated in IBD, with elevated levels of programmed cell death being observed in patients with both CD and UC [25, 26].

**Figure 1:**
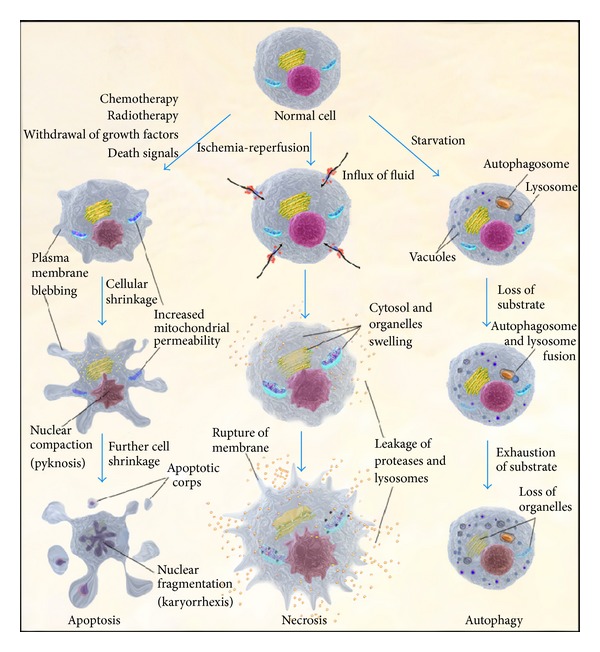
The three major pathways of cell death. Cells can be directed to different programmed cell death mechanisms depending on several factors. In the left, the apoptosis pathway is represented with the characteristic cellular shrinkage and formation of the apoptotic bodies without leakage of contents. In the middle, the necrotic pathway shows the cytosol and organelle swelling and rupture of plasma membrane with subsequent leakage of cellular contents. In the right, autophagy is illustrated with the appearance of vacuoles, the autophagosome, and its fusion with the lysosome, which ends in organelle digestion.

In IBD, all three types of programmed cell death are observed: apoptosis, autophagy, and necrosis (Figure 1). The exact programmed cell death pathway a cell undergoes depends on several factors such as the abundance of nutrients, the cell cycle stage, and the presence or absence of reactive oxygen species (ROS), adenosine triphosphate (ATP), autophagy protein 5 (ATG5), and nuclear factor kappa B (NF*κ*B) activation, among others [27–31].

## 2. Apoptosis

### 2.1. Intracellular Machinery of Apoptosis

Even though caspase-independent mechanisms mediated by the apoptosis-inducing factor (AIF) have been described, the activation of caspases is classically required to initiate the process of apoptosis [32]. Caspases comprise a specialized protease family, which contains a cysteine on the active site that cleaves the targets on their specific aspartic acid. Caspases not only participate in the progressive activation of other caspases but can also contribute to other processes such as the reduction of cell volume (pyknosis), chromatin condensation, nuclear fragmentation (karyorrhexis), and formation of plasma-membrane blebs [33, 34]. All these processes lead to alterations in cellular morphology resulting in cell and nucleus shrinkage without leakage of cellular content to the microenvironment. The intracellular machinery of apoptosis involves extrinsic and intrinsic pathways.

The extrinsic pathway also known as death receptor pathway involves the activation of death receptors, which are triggered by APO3L, TNF-*α*, FAS-L, and TNF-related apoptosis-inducing ligand (TRAIL). These ligands bind to their specific receptors such as APO3, TNF receptor (TNFR), FAS, and DR4/DR5 [3]. The ligand-receptor interaction initiates the destruction complex through the recruitment of intracellular adapted proteins called Fas associated with death domain (FADD) or TNF-*α* receptor-associated death domain (TRADD) that enables the catalytic activity of caspase-8, the central protease mediator of the extrinsic pathway [35].

The intrinsic pathway is observed when cells are under conditions such as DNA damage or growth factors withdrawal. In case of failure to repair the subsequent damage, the intracellular machinery stimulates the transcription of p53 [36, 37]. This gene, known as the guardian of the genome, stimulates other proteins such as p53 upregulated modulator of apoptosis (PUMA), Bcl-2 interacting mediator of cell death (BIM), and NOXA to initiate the cell death cascade [38–40]. The family of proteins that control the intrinsic pathway is known as Bcl-2. This family includes antiapoptotic and proapoptotic members. The difference between them lies in their homologous domains. The antiapoptotic members have four Bcl-2 homology regions and the proapoptotic members have three [41]. In addition, there is a third class of proapoptotic Bcl-2 family members that displays only the Bcl-2 homology 3 domain (“BH3-only”) [42].

In the intrinsic pathway, the balance between antiapoptotic and proapoptotic members is responsible for the determination of either cell death or cell recovery. When proapoptotic stimuli are prevalent, t-BID interacts with BAK and BAX leading to increased mitochondrial permeability and release of electron carrier protein cytochrome-c and SMAC/DIABLO. This protein inhibits the IAP, which are characterized by the blockage of caspase activity, while interacting with apoptotic protease activating factor 1 (Apaf-1) enabling the catalytic activity of caspase-9, the central protease mediator of the intrinsic pathway. Owing to the critical participation of mitochondria, this mechanism is also known as the mitochondrial pathway [35].

The activation of extrinsic (mediated by caspase-8) and intrinsic (mediated by caspase-9) pathways leads to activation of caspase-3, caspase-6, and caspase-7, which favors the cleavage of other proteins. A point of no return is achieved once the cell advances towards a critical state of destruction that will end in cell death and give rise to structures called apoptotic bodies.

### 2.2. Apoptosis in IBD

In the normal small intestine epithelium, all cells but Paneth cells and intestinal stem cells migrate from the base of the crypt to the villus tip where they are shed into the lumen. Bullen et al. studied almost 15.000 villus sections to closely determine the exact mechanisms behind cell shedding in the small intestine [43]. In this study, apoptotic cells were identified using antibodies against cleaved cytokeratin 18 and caspase-3. The authors found that cells always underwent apoptosis before shedding and that apoptotic bodies were never found in the epithelial monolayer. Interestingly, Marchiando et al. observed that morphologic changes typical of apoptosis were not apparent until the nucleus of the shedding cell had moved above the nuclei of adjacent cells, suggesting that, in the order of events, shedding leads to apoptosis [44]. The authors also demonstrated cleaved caspase-3 staining within the cytoplasm of shedding cells, which was only detectable after cell shedding was evident [44]. A broad-spectrum caspase inhibitor was then used and it was shown that almost all shedding events were blocked, indicating that caspase-3 cleavage is critical for cell shedding to occur [44].

In contrast, it has been shown that mice lacking caspase-3 (and caspase-8 and FADD as well) display limited apoptotic phenotype with no impact on gastrointestinal homeostasis [45–48]. In this regard, the activity of caspase-independent cell death pathways in the gut might be an important safeguard when caspase-mediated routes fail [32]. Interestingly, the early event in the cell shedding process seems to be the reorganization of ZO-1 and occludin which is accompanied by partial microvillus vesiculation and intracellular organelle breakdown, progressing to complete vesiculation of microvilli, nuclear condensation, and terminal contraction of surrounding epithelia [44].

Different from intestinal cell shedding, patterns of spontaneous apoptosis in the small and large intestine were more extensively described mostly due to the enterprise of the late Professor Christopher S. Potten [49]. Spontaneous apoptotic cells are restricted to the stem cell region in the small intestine and are seldom found in colonic crypts, being distributed along the length of the crypt [50]. This spontaneous apoptosis, which is p53-independent, has been seen as part of the stem cell homeostasis [49]. In contrast, Bcl-2 is minimally expressed in the small intestine, being more strongly expressed at the base of colonic crypts [50]. Interestingly, differences in Bcl-2 expression and cell death regulation can be accounted for the variability in tumor prevalence between the small and large intestines [50].

In IBD, high levels of apoptosis have been observed in the intestinal epithelium of patients. Our group investigated apoptosis in distinct mucosal compartments and the expression of Fas/Fas ligand in the inflamed and noninflamed intestinal mucosa of patients with IBD [51]. Colon specimens from patients with UC and CD were analyzed for densities and distribution of apoptotic cells determined by TUNEL essay. Colonic epithelium from patients with UC showed higher rates of apoptosis than controls, with no differences regarding CD [51]. Iwamoto et al. also found that apoptotic features were found in crypts of active UC, suggesting that loss of epithelial cells occurs mainly by apoptosis in involved intestine and also in adjacent uninvolved areas [52]. In keeping with these findings, Hagiwara et al. observed that the apoptotic indices in UC patients were significantly higher than those in controls but similar to those in infectious colitis patients [53]. Interestingly, apoptotic indices were significantly higher in patients undergoing surgery compared to those on medical treatment perhaps due to different disease severities [53].

In proteomic analysis, data also point towards the association between apoptosis and IBD. In this regard, in a small intestinal epithelial cell proteome study comparing CD, UC, and controls, 47% of all changes in the epithelial cell proteome were associated with signal transduction pathways, which included proapoptotic mechanisms [54]. In this study, the programmed cell death protein 8 (PDCD8) associated with caspase-independent apoptosis was almost 8-fold upregulated in inflamed versus noninflamed tissue regions in UC patients, supporting that programmed cell death mechanisms contribute to conditions of chronic inflammation in the gut [54]. As UC is mostly associated with a T helper type 2 (Th2) immune response, studies have suggested that Th2 cytokines might play a role in the enhanced apoptotic ratio found in the intestinal epithelium of patients with UC. In this regard, Rosen et al. observed that increased STAT6-dependent levels of IL-13 in UC were associated with greater epithelial cell apoptosis and barrier dysfunction and suggested that inhibition of STAT6 might decrease apoptosis in the epithelium of new-onset ulcerative colitis [55]. In accordance with these findings, IL-13 had a dose-dependent effect on transepithelial resistance of HT-29/B6 monolayers due to an increased number of apoptotic cells with parallel changes being observed in human samples [56].

Several animal studies further confirm the central role of apoptosis in disease mechanisms in IBD. The knockout mice for XBP1 (an endoplasmic reticulum (ER) stress-related transcription factor), for instance, develop spontaneous enteritis and are associated with Paneth cell dysfunction and subsequent apoptotic cell death [57]. More importantly, in humans, an association between UC and CD with XBP1 variants was identified and replicated as susceptibility genetic factors [57]. Likewise, NF-kappa B deficiency was shown to lead to apoptosis of colonic epithelial cells with subsequent impaired expression of antimicrobial peptides and translocation of bacteria into the mucosa [58, 59]. Another example is the conditional STAT3 knockout mice in intestinal epithelial cells; these animals were found to be highly susceptible to experimental colitis with important defects in epithelial restitution and enhanced apoptosis [60]. It has been further suggested that luminal nutrients and the microbiota can also influence the apoptotic ratio in the intestinal epithelium in mice. In this regard, luminal iron was shown to trigger epithelial cell stress-associated apoptosis through changes in microbial homeostasis [61]. In this study, in a CD-like ileitis mouse model, mice developed severe inflammation of the distal ileum with enhanced expression of proapoptotic cleaved caspase-3. Interestingly, absence of luminal iron sulfate reduced the expression of cleaved caspase-3 in the ileal epithelium [61].

In CD, the percentage of apoptotic enterocytes was found to be higher in involved compared to uninvolved areas and normal intestine, with no significant difference being found between uninvolved and normal mucosa [25]. These findings suggest that a greater apoptosis ratio in the intestinal epithelium of CD is associated with intestinal inflammation, being exclusively increased in inflamed areas [25]. Apoptosis was also observed after infection with several intestinal pathogens including* Salmonella*,* Shigella*, enteropathogenic* Escherichia coli*, human immunodeficiency virus type 1,* Helicobacter pylori*, and* Cryptosporidium parvum* [62]. In the case of infectious involvement of the intestine, pyroptosis, another form of cell death similar to apoptosis but less characterized, was also observed [63, 64]. This type of cell death forms a complex of proteins called inflammasome (or pyroptosome) that requires caspase-1 and activates interleukin-1 beta (IL-1*β*) and IL-18, two types of proinflammatory cytokines, which are predominant in T helper cells type 1 (Th1) immune responses [65].

**Figure 2:**
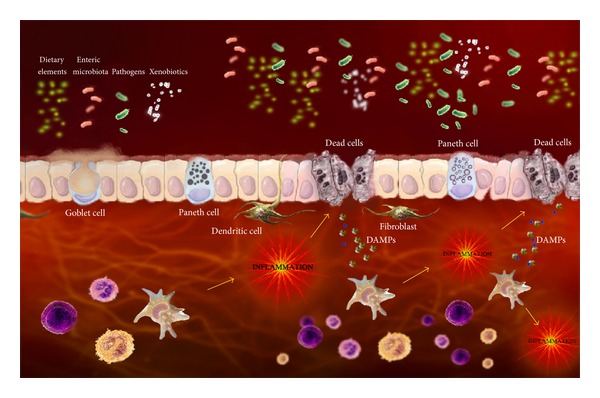
Simplified cartoon of the integrated intestinal homeostatic mechanisms showing the interplay between cell death and innate immunity in intestinal inflammation. Abnormal bacterial sensing through NOD-like and TLR in epithelial cells and dendritic cells in addition to Paneth cell dysfunction are greatly interrelated with the unfolded protein and autophagy pathways. The resulting production of chemokines and cytokines and the activation of immune cells in the lamina propria determine further epithelial barrier defects, with additional exposure to diverse intraluminal contents, enhanced by contact with damage-associated molecular patterns (DAMPs), in a self-perpetuating amplification loop. Figure adapted from Nunes et al. [5].

In the gut, inflammasome activation has been largely associated with the nucleotide-binding-oligomerization-domain- (NOD-) like receptors, which can sense bacterial components and also noninfectious elements regarded as damage-associated molecular pattern (DAMPS), molecules that can initiate and perpetuate immune response (Figure 2) [66]. In particular, NLRP3 is a NOD-like receptor that can be triggered by bacterial constituents and also by synthetic purine-like compounds, endogenous urate crystals, and exogenous adenosine triphosphate (ATP) [67]. Furthermore, it has been postulated that NLRP3 inflammasome activation can be mediated by pannexin-1 and P2X_7_ receptor, a member of the ATP-activated P2X purinergic receptors family [68]. The P2X_7_ receptors have been shown to function as danger sensors in immune cells and have been implicated in different biological functions, including apoptosis and the production and release of proinflammatory cytokines [69]. In addition, ATP was shown to induce apoptosis and autophagy in human epithelial cells, possibly via reactive oxygen species production [27]. These data, in conjunction with recent results from our group comprising experimental colitis [29] and human IBD [28], support the involvement of P2X_7_ receptors and the consequent inflammasome activation in the pathogenesis of IBD.

When it comes to response to therapy, polymorphisms in apoptosis genes were found to predict response to anti-TNF therapy in luminal and fistulizing CD [70]. In a cohort of 287 consecutive patients treated with infliximab, Fas ligand and caspase-9 genotypes predicted the outcomes after anti-TNF therapy. Interestingly, concomitant thiopurine therapy overcame the effect of unfavorable genotypes [70]. Regarding the effects of anti-TNF therapy on epithelial cells apoptosis, Zeissig et al. showed that, after anti-TNF treatment, a downregulation of epithelial apoptosis takes place in active CD [71]. In this study, the epithelial apoptotic ratio was increased in CD compared to controls and subsequently decreased after anti-TNF was introduced [71]. Marini et al. observed that anti-TNF therapy decreases the severity of murine CD-like ileitis by abolition of intestinal epithelial cell apoptosis [72]. In this study, a single injection of anti-TNF resulted in a marked suppression of intestinal inflammation, with a significant reduction in epithelial apoptosis. In contrast, an increase in lamina propria mononuclear cell apoptosis was observed. These results were confirmed in vivo by TUNEL staining, demonstrating that anti-TNF therapy involves homeostatic regulation of mucosal cell apoptosis [72].

## 3. Necrosis

### 3.1. Intracellular Machinery of Necrosis

Necrosis is derived from the Greek word “nekros” and means corpse [73]. To initiate the process of necrosis, the store of ATP is depleted by PARP (an enzyme which participates in DNA repair), which determines the shift from apoptotic to necrosis [74]. In the necrotic process, cell and organelles swell and rupture with subsequent leakage of cellular content to the microenvironment causing an inflammatory response. Until recently, cells were believed to passively undergo necrosis after external environment changes such as intestinal ischemia, inflammation, significant alterations in temperature, pH, and mechanical force [31, 75–77].

In the last two decades, however, several groups demonstrated that cells could undergo a necrosis-like cell death after TNF incubation [78–80]. Additional work described that this particular form of programmed cell death was triggered by death receptors and stimulated by caspase-8 inhibition [81, 82]. Because of its fine regulation, this cell death mechanism was posteriorly called necroptosis or programmed necrosis [83]. Necroptosis is characterized by the same morphologic features of necrosis as cell swelling, mitochondria dysfunction, membrane permeabilization, and release of cytoplasmic content, being also associated with high mitochondrial reactive oxygen species (ROS) production and it does not involve DNA fragmentation [84].

Necroptosis can be activated by lipopolysaccharides (LPS), physical-chemical stress, ionizing radiation, calcium overload, anticancer drugs, and DNA damage among other stimuli [84]. Signaling can be initiated through activation of members of the tumor necrosis factor (TNF) family and this pathway has been shown to be mediated by kinases receptor-interacting protein 1 (RIP1) and receptor-interacting protein 3 (RIP3) [47]. Upon induction of necrosis, RIP3 is recruited to RIP1 to establish a necroptosis inducing protein complex [47].

### 3.2. Necrosis in IBD

Cytotoxic bacteria were shown to induce necrosis in intestinal epithelial cells, which indicates that this cellular death process has an important role in infectious gastrointestinal diseases [85]. In CD, necrosis had been observed in electron and light microscopic of the intestinal epithelium [86]. In this study, samples from patients with CD, UC, and controls were evaluated. Patchy necrosis without acute inflammation was observed exclusively in patients with CD, indicating that this finding could have developed prior to inflammation [86].

Recently, two independent groups assessed the role of the programmed necrosis in IBD. Christoph Becker's group demonstrated the role of Caspase-8 in the regulation of necroptosis in the intestinal epithelium [47]. In this study, mice with a conditional deletion of caspase-8 in the intestinal epithelium spontaneously developed terminal ileitis and were highly susceptible to DSS colitis [47]. These mice also lacked Paneth cells, indicating dysregulated antimicrobial immune cell functions in the intestinal epithelium. In addition, epithelial cell death was induced by TNF-*α* and was associated with increased expression of RIP3 [47]. More importantly, the authors identified high levels of RIP3 in human Paneth cells and increased necroptosis in the terminal ileum of patients with CD, suggesting a potential role of necroptosis in the pathogenesis of this disease. In the other study, Welz et al. showed that knockout mice for FADD in intestinal epithelial cells spontaneously develop epithelial cell necrosis with loss of Paneth cells and small and large bowel inflammation [48]. In addition, MYD88 deficiency or elimination of microbiota prevented colon inflammation, indicating that toll-like receptor signaling drives the pathology in these animals [48].

## 4. Autophagy

### 4.1. Intracellular Machinery of Autophagy

Autophagy is derived from the Greek word that means “self-eating” [87]. This process is mainly known as the cell mechanism to recycle its own nonessential organelles, which can be activated by the lack of nutrients and growth factors in the extracellular microenvironment [88]. The characteristic structures of autophagy are the vacuoles, slight chromatin condensation, and the autophagosome, which fuses with lysosomes to digest material into substrates [87, 89]. The autophagosome is best visualized by electron microscopy and is composed of a double membrane lysosomal-derived vesicle that catabolizes the nonessentials or damaged particles and organelles [90]. The intracellular machinery of autophagy is composed of a complex of proteins formed by the class III phosphatidylinositol-3-kinase (PI3K), also known as Vps34, and the Bcl-2 interacting BH3 domain protein, Beclin-1 (BECN1). Both proteins are required for the autophagosome formation [91]. Signaling can be initiated through the mammalian target of rapamycin (mTOR) pathway, a serine/threonine kinase that participates in several mechanisms involved in cell survival.

Autophagy constitutes a self-degradation process, representing a critical mechanism for cytoprotection of eukaryotic cells. However, in the context of cancer, autophagy appears to play an ambiguous role. In association with apoptosis, autophagy can act as a tumor suppressor. On the other hand, defects in autophagy, in concert with abnormal apoptosis, may trigger tumorigenesis and also therapeutic resistance [92, 93].

### 4.2. Autophagy in IBD

#### 4.2.1. ATG16L1

A link between IBD and autophagy was first established when an association between CD and a single-nucleotide polymorphism (SNP) in the autophagy-related 16-like 1 gene (ATG16L1) was first reported by Hampe et al. [94] and later replicated by the same group [95]. This SNP (rs2241880) resulted in a threonine-to-alanine substitution at the amino acid position 300 of the protein (T300A) [94]. ATG16L1 is a central adaptor in the autophagosome formation. The rs2241880 variant is commonly found in the population, with 45–50% of healthy subjects carrying the polymorphism [96].

In the first study by Hampe et al., using haplotype and regression analysis, the authors found that the rs2241880 SNP carried all disease risk exerted by the ATG16L1 locus associated with CD in 3 European cohorts of CD patients [94]. Importantly, this association was not observed in a German cohort of UC cases, suggesting that the underlying biological process was specific to CD [94]. In their second study, the authors found that only individuals who were homozygous for the T300A-encoding variant of ATG16L1 were under higher risk to develop CD, suggesting a recessive model for the action of ATG16L1 [95]. In addition, a higher frequency of the rs2241880 allele was found in patients with ileum involvement, being the association with small bowel disease still significant even after adjustment for CARD15/NOD2 mutations [95]. This association with ileal involvement was confirmed by some [97] and could not be replicated by others [98]. A highest frequency of the rs2241880 SNP was also observed in individuals with childhood-onset CD [95] but others argue that these differences are driven by variations in disease location between late- and early-onset CD [97].

After the association between ATG16L1 polymorphisms with the development of CD was established, efforts were made to determine disease-related mechanisms, which could explain this specific susceptibility. Saitoh et al. generated ATG16L1 mutant mice and examined its function in autophagosome formation and the regulation of immune responses [99]. ATG16L1 mutant mice expressed deleted forms of the protein lacking the entire coiled-coil domain [99]. Most ATG16L1-deficient mice died within 1 day, indicating that the protein was required for neonatal survival [99]. In addition, in mouse embryonic fibroblasts (MEF) from ATG16L1-deficient mice, formation of autophagosomes under starved conditions was not observed, suggesting that ATG16L1 was essentially required for autophagy [99]. Furthermore, the authors examined the impact of ATG16L1 on cytokine production in response to lipopolysaccharide (LPS), showing that IL-1*β* and IL-18 were highly upregulated in ATG16L1-deficient cells compared with wild-type after toll-like receptor stimulus [99]. Cleaved caspase-1, an activated form that mediates processing of IL-1*β*, IL-18, and apoptosis, was also detected in the supernatants of ATG16L1-deficient macrophages following LPS stimulation. Importantly, these results indicated that toll-like receptor signaling is only associated with the formation of autophagosomes in nutrient-deprived macrophages [99]. In vivo, Saitoh et al. also observed that ATG16L1-deficiency exacerbates inflammation in DSS-induced colitis [99]. Chimeric mice with ATG16L1-deficient hematopoietic cells died due to acute weight loss and severe inflammation in the distal colon [99]. In these mice, serum levels of the proinflammatory cytokines IL-1*β* and IL-18 were significantly elevated and their mortality rate was improved after injection of neutralizing antibodies for these cytokines, indicating that autophagy might play a protective role in massive inflammation during acute colitis [99].

Cadwell et al. were the first to show that the ATFG16L1 protein was critical for the biology of Paneth cells [100]. In mice, ATG16L1- and ATG5-deficient Paneth cells exhibited notable abnormalities in the exocytosis pathway. In addition, ATG16L1-deficient Paneth cells had increased expression of genes involved in the lipid metabolism of acute phase reactants and adipocytokines [100]. In addition, CD patients who were homozygous for the ATG16L1 risk allele displayed Paneth cell abnormalities similar to those observed in ATFG16L1-deficient mice and expressed also increased levels of leptin [100]. Later, the same group also showed that ATFG16L1 deficiency alone was not enough for the development of Paneth cell abnormalities [101]. In this regard, mice housed at an enhanced barrier facility were similar to wild-type controls, failing to display the aberrant phenotype [101]. These results suggest that Paneth cell abnormalities associated with ATFG16L1 deficiency require an exogenous factor displayed in the microbiota of mice sitting at conventional animal facilities [101]. In the intestine, further studies also suggested that defects in the ATFG16L1 autophagy pathway are important in the presence of bacteria. Cooney et al. observed that NOD2 triggering induces autophagy in dendritic cells, which required ATG16L1, and that NOD2-mediated autophagy was necessary for CD4+ T cell responses in dendritic cells [102]. The relationship between NOD2 and ATG16L1 is not solely related to autophagy. Sorbara et al. have shown that knockdown of ATG16L1 expression specifically enhances NOD-driven cytokine production and that these findings also occurred in cells with an autophagy-incompetent truncated form of ATG16L1 [103].

Others also suggested that the impact of the ATG16L1 risk allele on CD might not be exclusively related to abnormalities in autophagy. Fujita et al., for instance, have shown that the T300A mutant has little impact on autophagy against* Salmonella*, proposing that this variant is differentially involved in CD and canonical autophagy [104]. In keeping with these findings, Messer el al. found that ATG16L1-deficient cells were resistant to cellular invasion by* Salmonella* [105]. Conway et al., however, demonstrated that autophagy was induced in small intestine and cecum of mice after Salmonella infection and this required ATG16L1 [106]. In this study,* Salmonella* colocalized with microtubule-associated protein 1 light chain 3*β* in the intestinal epithelium of control mice but not in mice lacking ATG16L1 in epithelial cells [106]. Consistent with these findings, these transgenic mice had increased inflammation and systemic translocation of bacteria compared with control animals. In this regard, autophagy is important for the maintenance of cellular homeostasis after infection, participating in the clearance of pathogens found in the ileum of CD patients [107].

Murthy et al. filled the gaps between autophagy, apoptosis, and inflammation, suggesting that the T300A variant causes sensitization to caspase-3-mediated cleavage of ATF16L1 [108]. The authors demonstrated that caspase-3 activation leads to accelerated degradation of ATG16L1 in the presence of the T300A variant. They propose that, in healthy intestine, the turnover of ATG16L1 is dependent on basal caspase-3 activity; in the presence of T300A, however, the persistence of apoptotic stimuli enhances ATG16L1 cleavage, triggering cytokine production and inflammation [108]. More recently, an association between autophagy and the ER stress response gene Xbp1 was shown to synergistically prevent ileal inflammation [109]. In this regard, Arthur Kaser's group has shown that Xbp1 loss in intestinal epithelial cells induced autophagy, most notably in Paneth cells, as a compensatory mechanism in intestinal epithelial cells upon sustained ER stress [109]. Mice with impaired ER stress signaling and autophagy developed transmural inflammation, characterized by acute and chronic inflammation extending to the muscularis propria and serosa, as fistulizing CD. This phenotype displays the important role of autophagy in the defense against ER stress in the intestinal epithelium [109]. This model is in keeping with recent data showing that ATG16L1 T300A polymorphisms define a specific subtype of patients with CD, characterized by Paneth cell ER stress even during quiescent disease [110].

#### 4.2.2. IRGM

Genome-wide association studies identified the autophagy gene IRGM on chromosome 5q33.1 to be strongly associated with CD [16] and to a lesser extent with UC [15, 111]. The IRGM gene belongs to immunity-related GTPases, a family of genes in mammalian species induced by interferons, though the human form seems to lack interferon-responsive elements [112, 113]. Two polymorphisms of IRGM have been strongly associated with CD, a silent tag-SNP variation within the coding region (c.313C>T) and a 20 kb deletion upstream of the IRGM gene [113–116]. In this regard, the coding-sequence variation was not thought to be the source of this association due to the absence of changes in IRGM protein structure [114, 115].

Brest et al. subsequently suggested that this synonymous variant (c.313C>T) was responsible for a disruption in a miRNA-binding site in individuals with the risk haplotype (T), resulting in lack of miRNA regulation in these patients [117]. In this regard, in subjects with CD, colonic epithelial cells have striking decreased IRGM levels only in patients homozygous for the protective IRGM haplotype (C), being the expression more reduced in inflamed tissue compared to involved mucosa in remission [117, 118]. These data suggest that lack of miRNA regulation and consequent overexpression of IRGM secondary to the risk allele (T) contribute to the association of this region with CD [118]. Importantly, overexpression of IRGM was associated with lower autophagy efficacy [117]. The other polymorphism, the 20 kb deletion upstream of IRGM, was first identified by McCarroll et al. in perfect linkage disequilibrium with the most strongly CD-associated SNP causing IRGM to segregate in a risk sequence (deletion present) and a protective sequence (deletion not present) [114]. Functionally, in this study, cells lacking IRGM have decreased proportion of internalized bacteria by autophagosome and overexpression of this molecule causes an increase in autophagy activity [114]. In summary, the current evidence suggests that differences in miRNA regulation or presence/absence of the upstream deletion sequence can affect IRGM expression leading to autophagy dysfunction.

Several studies tried to correlate variants in the IRGM gene with specific CD clinical features. In this regard, a large German study assessed the influence of the IRGM SNPs on disease phenotype, also evaluating interactions with other IBD susceptibility genes, particularly ATG16L1 [119]. In this study, based on the Montreal classification of IBD, none of the IRGM SNPs investigated were associated with specific disease features in CD or UC. In contrast, other studies found some associations between IRGM SNPs and clinical outcomes. Accordingly, IRGM SNPs were associated with fistulizing CD and perianal fistulas in a large cohort of Italian patients [120], with ileal involvement in subjects in New Zealand [121] and in Portugal [122] and with ileocolonic resection in a small cohort of American patients [123]. In addition, the IRGM CD risk variant was also associated with increased antiflagellin seropositivity [124] and a positive response to biologic therapy [122].

## 5. Conclusion

The representation of the different cell death pathways as individual and isolated mechanisms is entirely schematic and it does not reflect reality. A large and growing body of evidence has demonstrated that there is a dynamic crosstalk and much redundancy among different types of cell death mechanisms [125–127]. In this regard, it has been shown, for instance, that TNF-*α* treatment can induce either apoptosis or necrosis depending on the targeted cell type, environmental conditions, and magnitude of the cellular insult [125]. In addition, the death receptors FAS, TNFR2, TRAILR1, and TRAILR2, which are characteristically associated with apoptosis, might also induce necroptosis after caspase blockage or starvation [125]. Even the induction of p53 transcription and the Bcl-2 family of proteins have been associated with necrosis, being BAX and BAK required for mitochondrial dysfunction in response to necroptotic agonists [128]. As another example of this complex interplay among cell death pathways, studies have shown that apoptosis and autophagy are activated in response to metabolic stress and that both autophagy and apoptosis are induced in response to ER stress, with the increase in autophagy being a contributing factor to ER-induced apoptosis [129].

In the IBD field, nevertheless, most studies evaluate these pathways in the context of bowel inflammation as isolated cell death mechanisms. In the case of autophagy, at least two different genes were found to be related to IBD through genome-wide association studies [16]. In mechanistic studies in vivo (both humans and mice) and in vitro, with extensive use of novel animal models, potential roles for apoptosis and necroptosis in the pathogenesis of these diseases have been also suggested. Recent studies point towards the existence of a complex crosstalk between autophagy/apoptosis, microbe sensing, and enhanced ER stress in the epithelium in the pathogenesis of CD [108]. Exciting new data indicate that the ileal involvement in CD might be related to a disturbance in Paneth cell function, establishing a link between innate immunity, ER stress, and cell death [108, 109]. In addition, necroptosis, a relatively novel programmed necrosis-like pathway associated with TNF receptor activation, also seems to play a role in the pathogenesis of CD and in specific experimental models of intestinal inflammation [47, 48]. Moreover, a stress-inflammation amplification loop mediated by DAMPs has been directly associated with cell death in the intestinal mucosa in both experimental models and human IBD [28, 29]. The cell death history in IBD seems to be an interesting example of data coming from huge hypothesis-free GWAS studies leading to hypothesis-driven mechanistic discoveries.
